# Pigmented villous nodular synovitis mimicking metastases on ^18^F-FDG PET/CT in a patient with rectal mucosal melanoma: a case report

**DOI:** 10.1186/s12891-019-3034-x

**Published:** 2020-01-08

**Authors:** Yu-An Yen, Li-Chun Wu, Na-Mi Lu, Chiang Hsuan Lee

**Affiliations:** 1 Department of Nuclear Medicine, Chi Mei Medical Center, 901, Zhonghua Rd., Yongkang Dist, Tainan City, 710 Taiwan; 2 Department of Pathology, Liouying Chi Mei Hospital, Tainan, Taiwan

**Keywords:** Melanoma, Pigmented villous nodular synovitis (PVNS), Positron emission tomography (PET), Rectum

## Abstract

**Background:**

Mucosal melanomas are rare and have a high potential for metastasizing. Surgical resection is the treatment of choice for single distant metastases. Malignant melanoma usually shows the highest uptake of fluorine-18 fluorodeoxyglucose (^18^F-FDG). ^18^F- FDG positron emission tomography /computed tomography (PET/CT) is usually used for melanoma staging. An extensive literature review revealed only 4 published case reports and an original paper involving 8 cases (12 cases in total) of patients with skin melanomas in whom pigmented villous nodular synovitis (PVNS) mimicked metastatic melanoma, however, none of the melanomas reported were of rectal mucosal origin.

**Case presentation:**

A 60-year-old woman presented with recent diagnosis of rectal mucosal melanoma, two additional ^18^F-FDG-avid lesions in the left ankle and left foot were detected on ^18^F-FDG PET/CT. Metastases were initially suspected; however, the final diagnosis was PVNS.

**Conclusions:**

This is the first report of PVNS mimicking metastases on ^18^F-FDG PET/CT in a patient with rectal mucosal melanoma. Although high ^18^F-FDG-avid lesions in patients with rectal mucosal melanoma are highly suspected to be metastasis and warrant an meticulous examination, the present case is a reminder that in such patients, not all lesions with high ^18^F-FDG uptake, especially those near a joint, are metastases and that more extensive resection is unnecessary.

## Background

Only 1% of all melanomas arise from mucosa; most melanomas arise from skin [1]. Mucosal melanomas arise primarily in the head and neck, anorectal, and vulvovaginal regions. Of all colorectal malignancies, anorectal mucosal melanomas are rare (0.05%) [2], and they have a high potential for metastasis [1, 3, 4]. Surgical resection of the primary tumor, performed by wide local excision, is the mainstay of treatment.

Of all cancers, malignant melanoma usually shows the highest uptake of fluorine-18 fluorodeoxyglucose (^18^F-FDG) [5]. Positron emission tomography/computed tomography (PET/CT) with ^18^F-FDG is a highly effective way to screen for metastases of malignant melanoma throughout the body [6–8]. ^18^F-FDG PET/CT can reveal unexpected metastases, often outside the field of view of the other imaging modalities; such findings necessitate a change in patient management [9]. Surgical excision of metastases is recommended if only one or a few sites of disease are apparent [10].

However, not all highly ^18^F-FDG-avid lesions are malignant. Benign conditions and lesions can have high ^18^F-FDG uptake, including hyperplasia, benign tumors, and any inflammation or infection [11, 12]. Therefore, lesions should be histologically confirmed, particularly when PET/CT findings might prompt a change of treatment.

Pigmented villous nodular synovitis (PVNS) represents an uncommon benign proliferative process characterized by focal or diffuse hyperplasia of synovial villi that affects the synovial joints, tendon sheaths, and bursa membranes. In asymptomatic cases, no additional treatment is required [13–16]. The knee, followed by the hip, is the most common location of PVNS. Other large joints affected include the ankle, the shoulder, and the elbow, in decreasing order of frequency. PVNS lesions have high ^18^F-FDG uptake and are known to have a false-positive appearance on ^18^F-FDG PET/CT [17, 18].

We describe the case of a patient with rectal mucosal melanoma and two additional ^18^F-FDG-avid lesions, one in the left ankle and one in the left foot. These lesions were detected on ^18^F-FDG PET/CT and initially suspected to be metastases, but the final diagnosis was PVNS. To our knowledge, this is the first report of PVNS that mimics metastases on ^18^F-FDG PET/CT in a patient with rectal mucosal melanoma.

## Case presentation

**Fig. 1:**
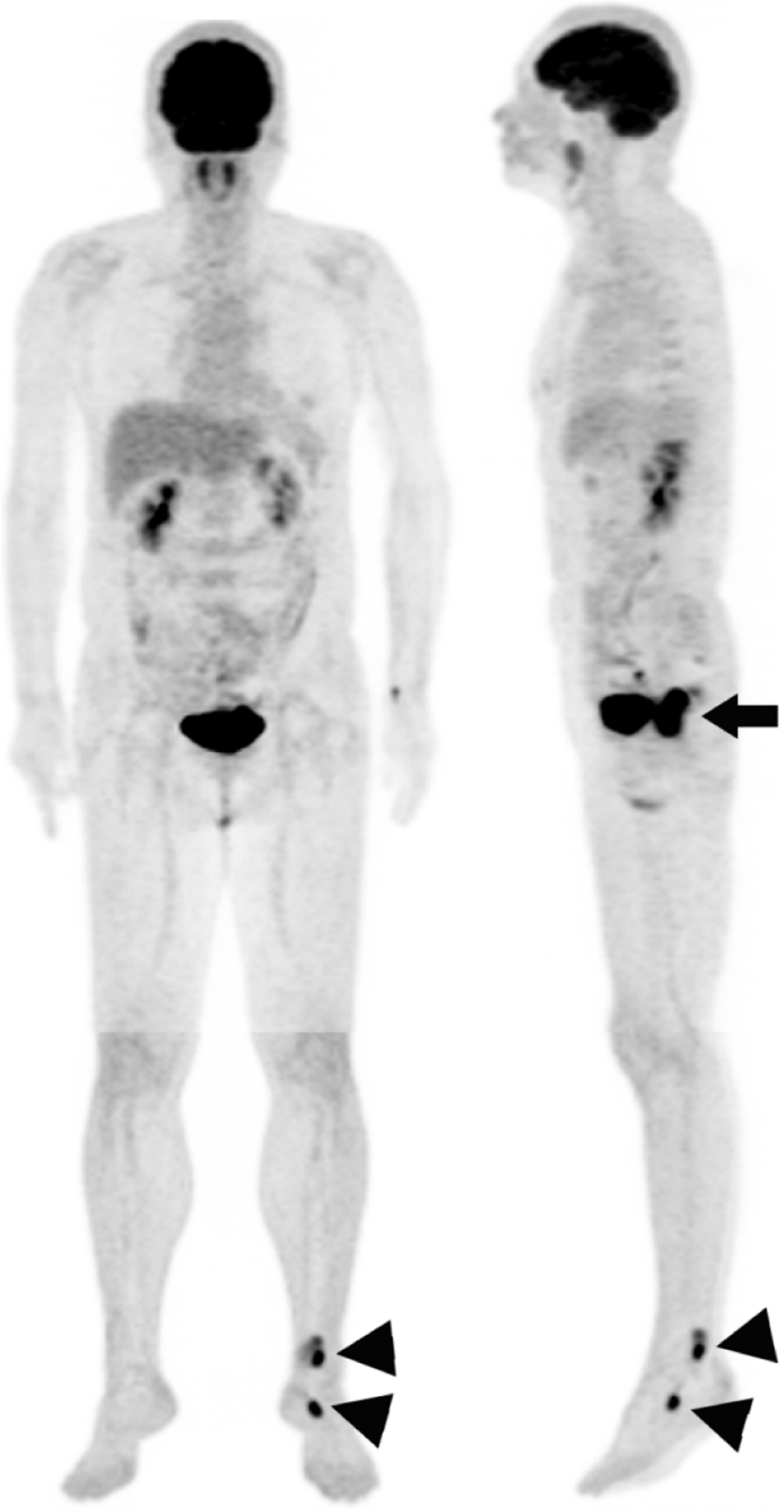
Anterior (left) and left lateral (right) whole-body Fluorine-18 fluorodeoxyglucose (^18^F-FDG) maximum-intensity projections in a patient with rectal melanoma. An intensely ^18^F-FDG-avid lesion was present in the rectum (arrow) and two additional intensely ^18^F-FDG-avid lesions were present in the left ankle and in the left foot (arrowheads)

**Fig. 2:**
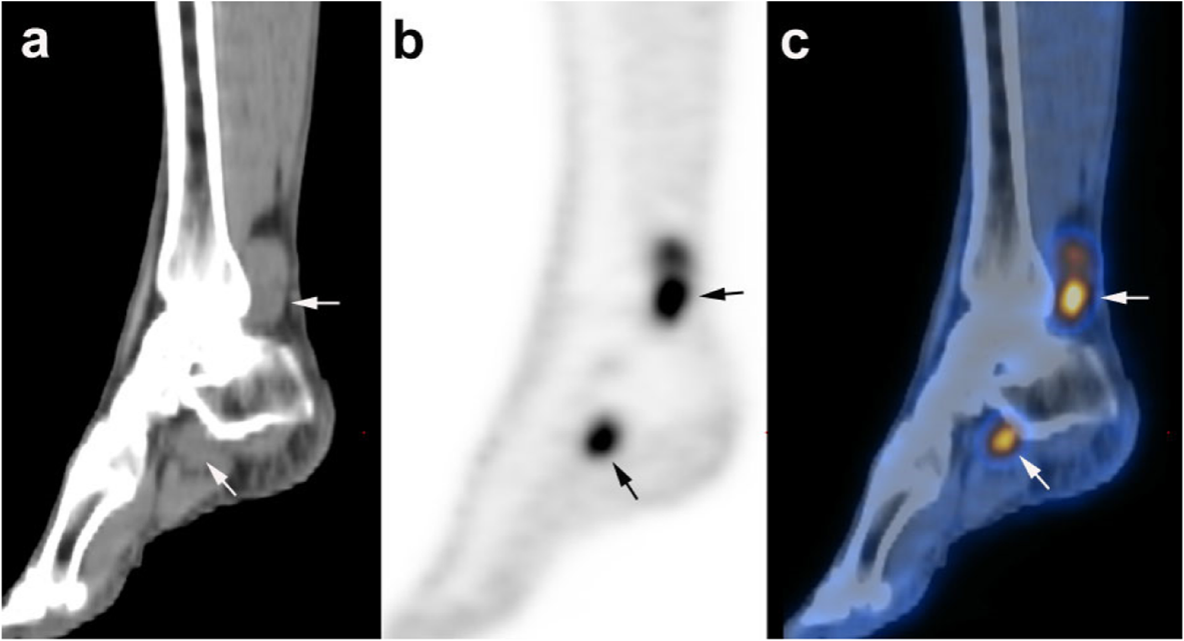
Sagittal positron emission tomography (PET; **a**), computed tomography (CT; **b**), and fused Fluorine-18 fluorodeoxyglucose (^18^F-FDG) PET/CT (**c**) of the patient’s left ankle and left foot. Two ^18^F-FDG-avid lesions were present in these locations beside the flexor hallucis longus muscle and tendon (arrows)

A 60-year-old woman presented to her primary care physician with bloody stool for 2 months. Laboratory examination revealed Hb and Hct levels were 11.8 g/dL and 34.2%, (reference range: 11.6–14.8 g/dL and 34–44%), respectively. Stool occult blood was < 7 ng/mL (reference range: < 12 ng/mL). Physiological examination did not reveal any other skin lesion that could be suspected for melanoma. Colonoscopy and biopsy performed at another hospital revealed a malignant melanoma at the anorectal site; thereafter, the patient was transferred to our hospital only with pathology report for further management. Routine ^18^F-FDG PET/CT examination performed after the biopsy for melanoma staging revealed a highly ^18^F-FDG-avid lesion in the rectum (Fig. 1). The maximum standardized uptake value (SUV_max_) was 15.3. Two additional high ^18^F-FDG-avid lesions were found in her left ankle and left foot (SUV_max_, 8.9; Fig. 2).

The lesion with the highest ^18^F-FDG avidity noted was diagnosed as rectal melanoma; moreover, the lesions in the left ankle and the left foot showed high ^18^F-FDG avidity. For these reasons, and because melanoma has a high metastatic potential, these lesions were first thought to be distant metastases.

**Fig. 3:**
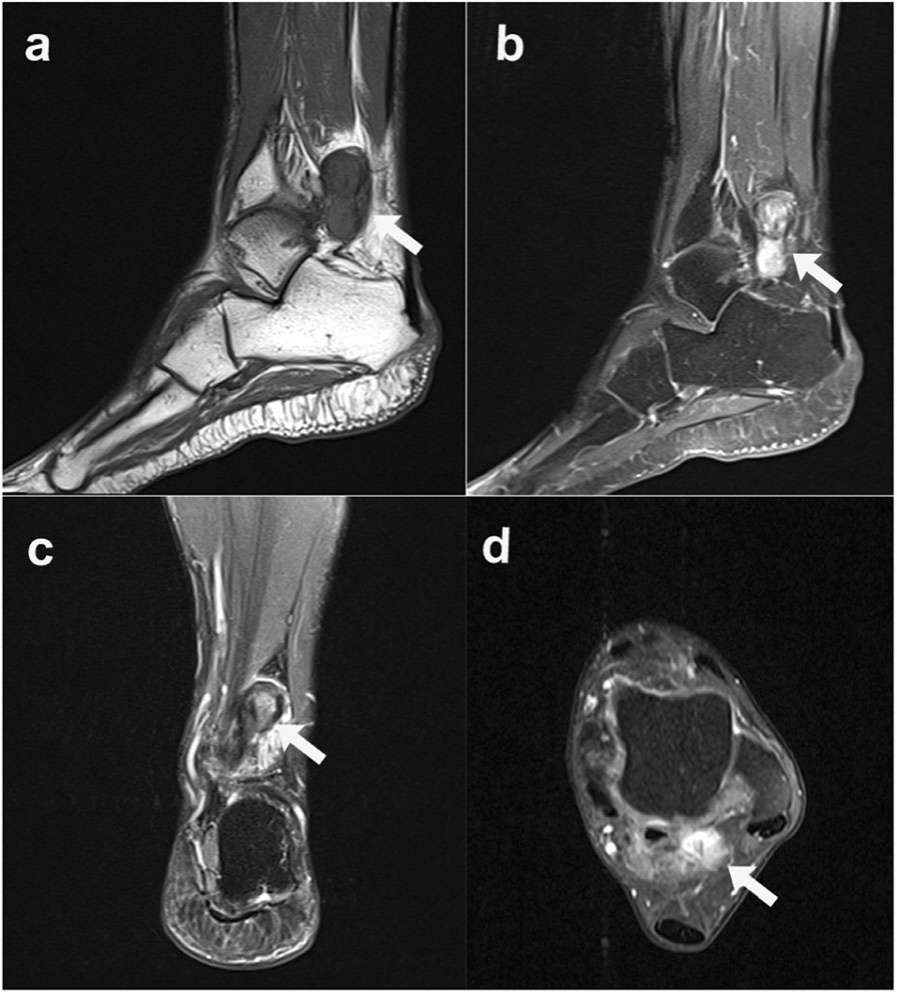
Magnetic resonance images of the patient’s left ankle. **a** Sagittal T1-weighted turbo spin echo (TSE) image. **b** Sagittal T1-weighted TSE contrast-enhanced image with Ga-DTPA fat -suppressed. **c** Coronal proton density (PD)-weighted TSE fat-suppressed image. **d** Transverse T1-weighted TSE contrast-enhanced image with Ga-DTPA fat-suppressed. A well-enhanced mass was identified beside the flexor hallucis longus muscle at the level of the ankle joint (arrows). Considering that the patient was diagnosed with melanoma, these appearances were in favor of metastases from melanoma according to a radiologist. However, the differential diagnosis was pigmented villous nodular synovitis

**Fig. 4:**
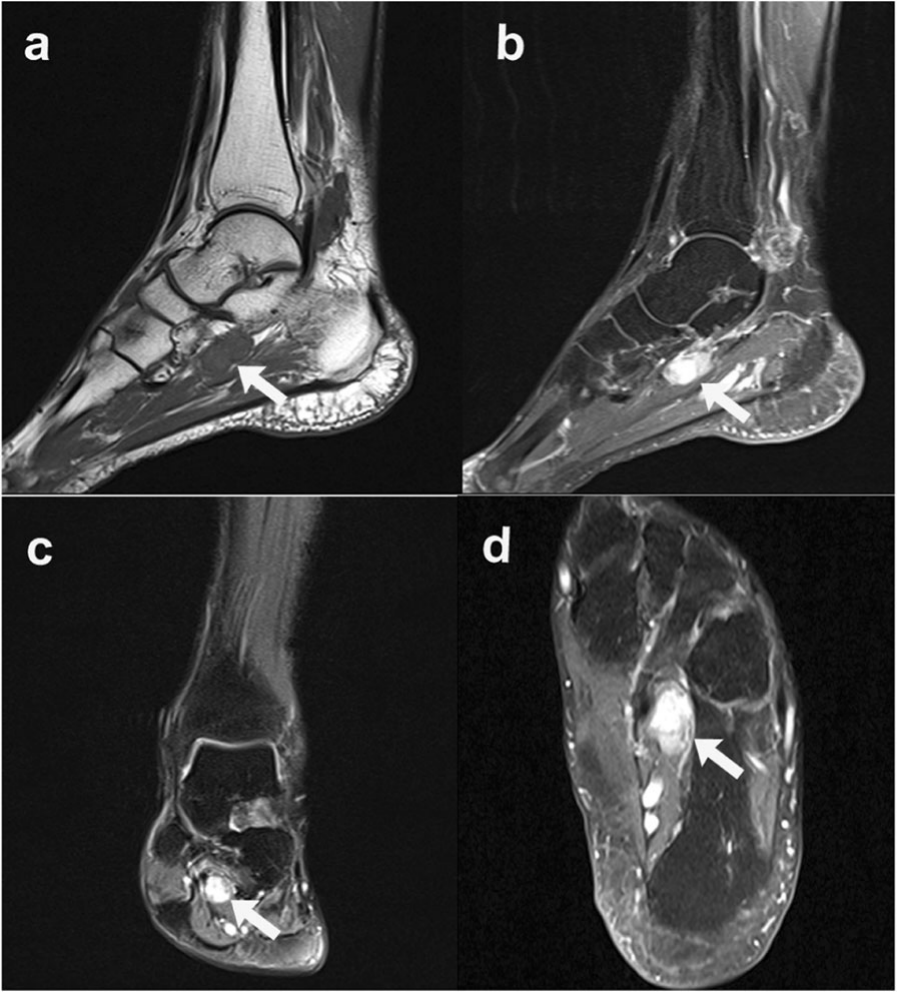
Magnetic resonance images of the patient’s left foot. **a** Sagittal T1-weighted turbo spin echo (TSE) image. **b** Sagittal T1-weighted TSE contrast-enhanced image with Ga-DTPA fat -suppressed. **c** Coronal proton density (PD)-weighted TSE fat-suppressed image. **d** Transverse T1-weighted TSE contrast-enhanced image with Ga-DTPA fat-suppression. Another well-enhanced mass was identified beside the flexor hallucis longus tendon at the level of the talonavicular joint (arrows). Considering that the patient was diagnosed with melanoma, these appearances were in favor of metastases from melanoma according to a radiologist. However, the differential diagnosis was pigmented villous nodular synovitis

**Fig. 5:**
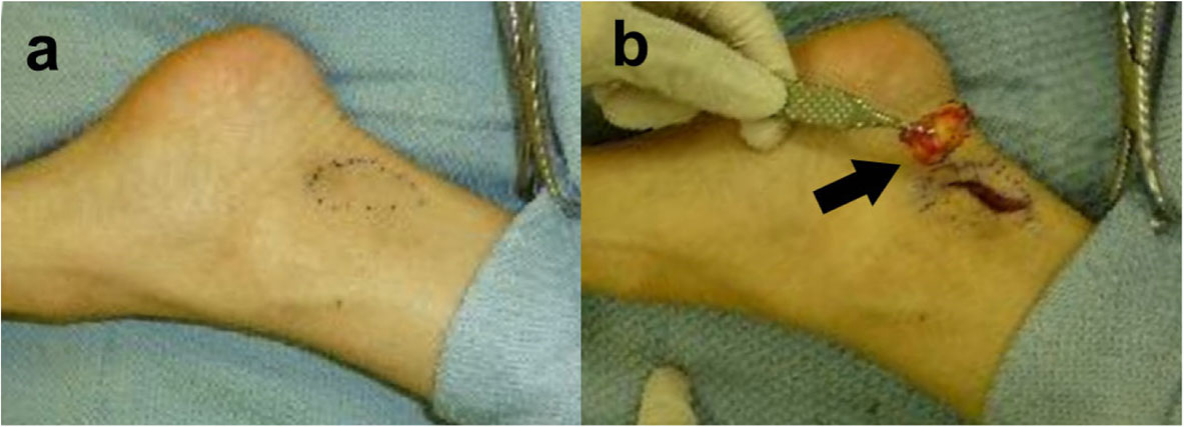
Excision of the tumor in the left ankle. **a** The location was marked (arrow) before surgery. **b** The mass (arrow) was removed

**Fig. 6:**
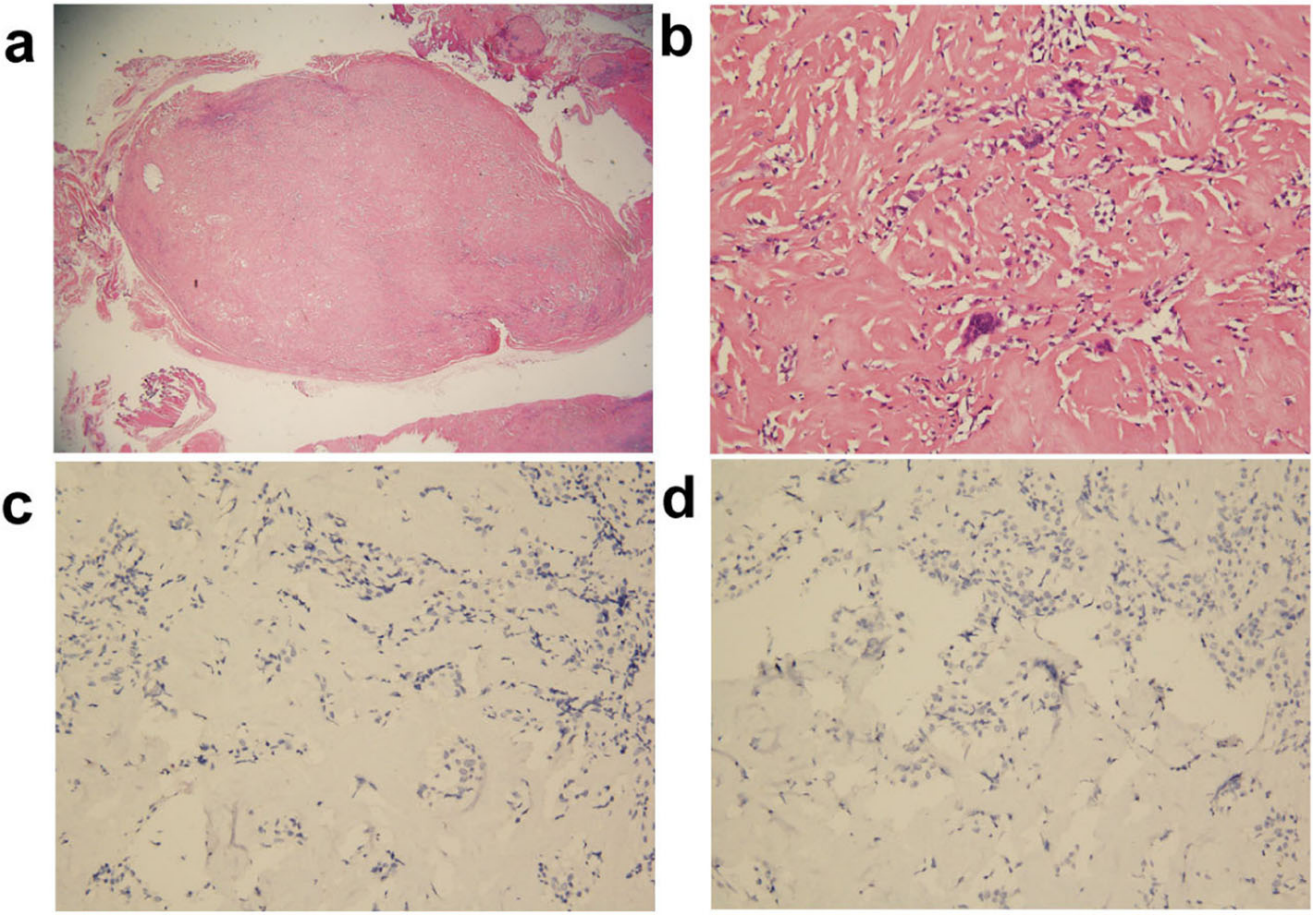
Pathological appearances of the mass from the left ankle. **a** Low-power view of the hypocellular, ovoid shaped tumor. **b** Focal areas had increased cellular infiltrates consisting of lymphocytes, macrophages, and multinucleated giant cells, surrounded by highly collagenized stroma. Special staining with Melan-A (**c**) and HMB45 (**d**), both of which are markers of melanoma, yielded negative results. The pathologically proved diagnosis was pigmented villous nodular synovitis (PVNS)

The patient was referred to a surgical oncologist to undergo additional workup. Magnetic resonance images (MRI), sagittal T1-weighted turbo spin echo (TSE) image (Figs. 3 and 4a), sagittal T1-weighted TSE fat-suppressed with Ga-DTPA contrast -enhanced image with Ga-DTPA fat suppression (Figs. 3 and 4b), coronal proton density (PD)-weighted TSE fat -suppressed image (Figs. 3 and 4c), and transverse T1-weighted TSE fat-suppressed with contrast-enhanced image with Ga-DTPA fat suppression (Figs. 3 and 4d) of the left foot, obtained to further characterize the lesions, revealed the two masses beside the flexor hallucis longus muscle and tendon, which were locations suggestive of metastases from melanoma; however, the differential diagnosis included PVNS. Out of concern that they represented metastatic melanoma, she was taken to the operating room. Surgery on the left ankle revealed firm, yellowish subcutaneous tumors beneath the deep fascia and the Achilles tendon. Intraoperative pathological study revealed that the lesion in the left foot was benign; therefore, only the mass in the left ankle was resected (Fig. 5), and special pathological staining revealed that the correct diagnosis was PVNS (Fig. 6). Thus, this patient underwent abdominoperineal resection. However, a follow-up abdominal CT conducted 3 months later revealed new multiple liver metastases, and chemotherapy treatment was initiated. Fortunately, a follow-up abdominal CT conducted 2 years later revealed that the liver lesions were nearly in complete regression. The patient is alive, and after another 2 years after complete regression, the patient is stable at present.

## Discussion and conclusion

Because anorectal melanomas have a high metastatic potential and high ^18^F-FDG uptake, any distant lesion that is highly ^18^F-FDG-avid is suspected as being a possible metastasis, and for any resectable ^18^F-FDG-avid lesions that are identified, aggressive surgical management is presumably necessary. However, in the present case, intraoperative pathological tests revealed benign tumor. Therefore, only lesion excision was performed.

An extensive literature review revealed only four published case reports and an original paper about 8 cases (12 cases in total) of patients with skin melanomas in whom PVNS mimicked metastatic melanoma, but none of the melanomas were mucosal [18–22]. To our knowledge, our case is the first report of PVNS mimicking metastases on ^18^F-FDG PET/CT in a patient with rectal mucosal melanoma.

In retrospect, there can be two indications suggesting that these lesions are not metastases from melanoma. First, we noted that although the lesions in the left ankle and left foot showed high ^18^F-FDG uptake (SUV_max_, 8.9), the uptake was moderately lower than that of the rectal melanoma (SUV_max_, 15.3). This suggests that these lesions were not the highest ^18^F-FDG-avid lesions. Second, the lesions was located on the ankle and foot, which are sites preceded by the knee and hip as the most common sites; therefore, the locations of the lesions were not at the most common sites of PVNS.

Because the treatment of PVNS differs significantly from that of metastatic melanoma, PVNS should be included in the differential diagnosis of melanoma, especially in cases when ^18^F-FDG PET/CT identifies possible metastatic spread near the joints, as in this case (in the left ankle and the left foot).

Although any highly ^18^F-FDG-avid lesion in patients with rectal mucosal melanoma is high suspect for being a metastasis and warrants an aggressive workup, some benign conditions and lesions can have high ^18^F-FDG uptake. This case is a reminder that not all lesions with high ^18^F-FDG uptake, especially not extremely high ^18^F-FDG uptake and near the joint, are metastases and that more extensive resection may not be necessary.

## Data Availability

Not applicable.

## Abbreviations

^18^F: Fluorine-18
FDG: Fluorodeoxyglucose
PET/CT: Positron emission tomography /computed tomography
PVNS: Pigmented villous nodular synovitis
SUV_max_: The maximum standardized uptake value

## References

[1] Chang AE, Karnell LH, Menck HR (1998). The National Cancer Data Base report on cutaneous and noncutaneous melanoma: a summary of 84,836 cases from the past decade. The American College of Surgeons Commission on Cancer and the American Cancer Society. Cancer.

[2] Cagir B, Whiteford MH, Topham A, Rakinic J, Fry RD (1999). Changing epidemiology of anorectal melanoma. Dis Colon Rectum.

[3] Iddings DM, Fleisig AJ, Chen SL, Faries MB, Morton DL (2010). Practice patterns and outcomes for anorectal melanoma in the USA, reviewing three decades of treatment: is more extensive surgical resection beneficial in all patients?. Ann Surg Oncol.

[4] Pessaux P, Pocard M, Elias D, Duvillard P, Avril MF, Zimmerman P, Lasser P (2004). Surgical management of primary anorectal melanoma. Br J Surg.

[5] Wahl RL, Hutchins GD, Buchsbaum DJ, Liebert M, Grossman HB, Fisher S (1991). 18F-2-deoxy-2-fluoro-D-glucose uptake into human tumor xenografts. Feasibility studies for cancer imaging with positron-emission tomography. Cancer.

[6] Mettler FA (2019). MJG: Hybrid PET/CT Neoplasm Imaging. Essentials of Nuclear Medicine and Molecular Imaging.

[7] Perng P, Marcus C, Subramaniam RM (2015). (18) F-FDG PET/CT and melanoma: staging, immune modulation and mutation-targeted therapy assessment, and prognosis. AJR Am J Roentgenol.

[8] Schwenzer NF, Pfannenberg AC (2015). PET/CT, MR, and PET/MR in lymphoma and melanoma. Semin Nucl Med.

[9] Gulec SA, Faries MB, Lee CC, Kirgan D, Glass C, Morton DL, Essner R (2003). The role of fluorine-18 deoxyglucose positron emission tomography in the management of patients with metastatic melanoma: impact on surgical decision making. Clin Nucl Med.

[10] Finkelstein SE, Carrasquillo JA, Hoffman JM, Galen B, Choyke P, White DE, Rosenberg SA, Sherry RM (2004). A prospective analysis of positron emission tomography and conventional imaging for detection of stage IV metastatic melanoma in patients undergoing metastasectomy. Ann Surg Oncol.

[11] Mettler FA (2019). MJG: Hybrid PET/CT Neoplasm Imaging. Essentials of Nuclear Medicine and Molecular Imaging.

[12] Harvey A, JPOM Z, James H, Fahey FH (2014). Thrall: Oncology: Positron Emission Tomography. Nuclear Medicine: The Requisites.

[13] Auregan JC, Klouche S, Bohu Y, Lefevre N, Herman S, Hardy P (2014). Treatment of pigmented villonodular synovitis of the knee. Arthroscopy.

[14] Korim MT, Clarke DR, Allen PE, Richards CJ, Ashford RU (2014). Clinical and oncological outcomes after surgical excision of pigmented villonodular synovitis at the foot and ankle. Foot Ankle Surg.

[15] Murphey MD, Rhee JH, Lewis RB, Fanburg-Smith JC, Flemming DJ, Walker EA (2008). Pigmented villonodular synovitis: radiologic-pathologic correlation. Radiographics.

[16] Tritschler P, Baudrez V, Mutijima E (2018). Diffuse pigmented Villonodular Synovitis of the Subtalar joint. J Belg Soc Radiol.

[17] Amber IB, Clark BJ, Greene GS. Pigmented villonodular synovitis: dedicated PET imaging findings. BMJ Case Rep. 2013;bcr2013009401:1–3. 10.1136/bcr-2013-009401 PMC3645251 23598941

[18] Broski SM, Murdoch NM, Skinner JA, Wenger DE (2016). Pigmented Villonodular Synovitis: potential pitfall on oncologic 18F-FDG PET/CT. Clin Nucl Med.

[19] Kitapci MT, Coleman RE (2003). Incidental detection of pigmented villonodular synovitis on FDG PET. Clin Nucl Med.

[20] Mahmood S, de Llano SR (2007). Localized nodular synovitis mimicking metastatic melanoma in a patient with metastatic melanoma on whole-body F-18 FDG PET/CT with MRI and pathological correlation. Clin Nucl Med.

[21] Selby L, Kukar M, Wang J, Beg M, Sullivan J (2014). Pigmented villous nodular synovitis mimicking metastatic melanoma on PET-CT. Int J Surg Case Rep.

[22] Wang S, Stewart JM, Ross MI, Prieto VG (2003). Extensive pigmented villonodular synovitis with markedly pigmented lymphadenopathy and its implication for differential diagnosis with malignant melanoma. Ann Diagn Pathol.

